# EEG-TNet: An End-To-End Brain Computer Interface Framework for Mental Workload Estimation

**DOI:** 10.3389/fnins.2022.869522

**Published:** 2022-04-25

**Authors:** Chaojie Fan, Jin Hu, Shufang Huang, Yong Peng, Sam Kwong

**Affiliations:** ^1^Key Laboratory of Traffic Safety on Track of Ministry of Education, School of Traffic and Transportation Engineering, Central South University, Changsha, China; ^2^Department of Computer Science, City University of Hong Kong, Kowloon, Hong Kong SAR, China; ^3^Hunan Communications Research Institute Co., Ltd., Hunan Communication & Water Conservancy Group Ltd., Changsha, China; ^4^School of Business and Trade, Hunan Industry Polytechnic, Changsha, China

**Keywords:** mental workload, brain computer interface, deep neural network, occupational safety, ergonomics

## Abstract

The mental workload (MWL) of different occupational groups' workers is the main and direct factor of unsafe behavior, which may cause serious accidents. One of the new and useful technologies to estimate MWL is the Brain computer interface (BCI) based on EEG signals, which is regarded as the gold standard of cognitive status. However, estimation systems involving handcrafted EEG features are time-consuming and unsuitable to apply in real-time. The purpose of this study was to propose an end-to-end BCI framework for MWL estimation. First, a new automated data preprocessing method was proposed to remove the artifact without human interference. Then a new neural network structure named EEG-TNet was designed to extract both the temporal and frequency information from the original EEG. Furthermore, two types of experiments and ablation studies were performed to prove the effectiveness of this model. In the subject-dependent experiment, the estimation accuracy of dual-task estimation (No task vs. TASK) and triple-task estimation (Lo vs. Mi vs. Hi) reached 99.82 and 99.21%, respectively. In contrast, the accuracy of different tasks reached 82.78 and 66.83% in subject-independent experiments. Additionally, the ablation studies proved that preprocessing method and network structure had significant contributions to estimation MWL. The proposed method is convenient without any human intervention and outperforms other related studies, which becomes an effective way to reduce human factor risks.

## 1. Introduction

Information systems are increasingly approaching the boundaries of human competence due to their increasing complexity and autonomy. A dynamic and automated adaptation of the system to the user state is required to minimize user overload in high-demand scenarios (Mühl et al., 2014). A reliable understanding of the user's current status, particularly the workload, is essential for timely and appropriate system adaptation (van Erp et al., 2010). The workload is a direct factor in unsafe operations. Workers of special occupational groups such as construction workers, car drivers, pilots are prone to physical exhaustion and lack of consciousness under high workloads for a long time, leading to numbness of safety conditions and causing great insecurity. Therefore, it is extremely important to effectively assess and reduce the workload of operators in preventing unsafe behaviors and reducing dangerous accidents. Thus, workload estimation is an actively growing research field, for it possesses numerous human factor applications in many occupational groups to reduce safety risks (Roy et al., 2016; Yin et al., 2019).

The workload is mainly divided into physical workload and mental workload (MWL). When the human body is under different physical workloads, various physiological parameters such as oxygen consumption, heart rate, pulmonary ventilation, energy expenditure rate, and various chemical enzymes related to energy conversion show changes. (Roscoe, 1992; Abdelhamid and Everett, 2002). However, the estimation of MWL is more complicated than physical workload, while the former is more closely associated with safety.

There are two types of MWL estimation methods, subjective and objective estimation methods (Hogervorst et al., 2014; Charles and Nixon, 2019). The subjective test is a self-recorded and a questionnaire-based test in which the subject's workload is scored. Among a large number of subjective estimation methods, the National Aeronautics and Space Administration's Task Load Index (NASA TLX) (Hart, 2006) and Subjective Workload Assessment Technique (SWAT) (Reid and Nygren, 1988) are the most popular subjective estimation methods. Additionally, the objective estimation methods are used to estimate their workload by collecting the object's physiological signals.

The objective test has developed rapidly in recent years due to developments in sensor technology. The rationality for the objective test based on physiological signals is that when people are under MWL, the parameters of each physiological condition deviate from the normal state. Thus, it is possible to detect changes in the body's physiological signals to estimate MWL. The changed physiological parameters include cardiac activity, electrical brain activity, eye movements, and metabolic changes (Fairclough and Houston, 2004). Therefore, many physiological indicators have been used to estimate MWL, such as electrocardiograms (ECG), eye movements, electroencephalography (EEG) measurements, respiration, and electromyography (EMG). Among these physiological indicators, EEG is widely used because MWL changes are closely linked to brain cortical activity and because it is non-smooth, non-invasive, and highly discriminative (Wilson et al., 1994; Dehais et al., 2020; Pieper et al., 2021; Liu et al., 2022; Yu et al., 2022). This is why EEG is also known as the gold standard. In conclusion, EEG had the best and most reliable estimation performance of MWL.

To sum up, in terms of accuracy and practicality, EEG is optimal for estimating the MWL. The entire framework also can be referred to as a brain-computer interface (BCI) by means of computer algorithms that decode information from the brain and thus access the state of the human. In this study, an end-to-end BCI framework using EEG is proposed to estimate workers' MWL continuously, which can directly decode EEG without feature extraction.

### 1.1. Related Study

#### 1.1.1. Handcrafted Features-Based BCI Framework

Brain-computer interface, as a new human-computer interaction technology, provides a new method of communication with the outside world and enables direct human control of machines. In recent years, with deep cross-fertilization of artificial intelligence technology in neuroscience, cybernetics, computer science, and other related fields, research on BCI cognitive status computing systems based on EEG. There are a large number of BCI frameworks for MWL assessment have been proposed in recent years, and most of the research has used handcrafted features. Lim et al. (2018) assessed the MWL induced by the single-session simultaneous capacity (SIMKAP) experiment. They collected the 14-channels of EEG and extracted different bands' power spectral density (PSD). The Neighborhood Component Analysis (NCA) was used to select critical features and the Support Vector Regression (SVR) model was trained to assess the MWL. A helmet with EEG sensors was designed by Wang et al. (2017) to meet the requirements of the construction industry, and they designed different construction activities to induce different levels of MWL. The results showed that Gamma waves and Fp1 and Tp10 channels are good candidates for MWL estimation in the frequency domain. However, the unavoidable limitations of current BCI frameworks which use handcrafted features should not be ignored. The extraction of EEG signal features requires researchers to master interdisciplinary theories and research results in stochastic signal analysis and cognitive neuroscience, raising the threshold for researching this field (Cheng et al., 2022). Thus, the incomprehensibility of domain knowledge can limit the extracted features that cannot effectively represent the implicit MWL-related information in the original signal. In addition, restricted by the performance of computing units of wearable devices, algorithms with high computational complexity cannot be applied on brain-computer interface systems. The computation of EEG features, especially non-linear features such as entropy value and complexity, requires much time and, thus, cannot meet the needs of brain-computer interface systems.

#### 1.1.2. Deep Learning-Based BCI Framework

To solve the feature extraction problem, inspired by the success of the feature extraction ability of convolutional neural network, decoding EEG according to CNN, which constructing the end-to-end BCI framework are receiving increasing attention. There are some related end-to-end studies in the field of EEG-based BCI frameworks for other tasks, such as emotion recognition, word imagined, and epileptic seizure recognition (Xu et al., 2020; Datta and Boulgouris, 2021; Hu et al., 2021). Furthermore, unlike the images, EEG signals are typically time-series signals, and the evolutionary trends in neural activity during complex or simple cognitive processing are of equal interest. Therefore, combined models by merging CNN and Long short-term memory (LSTM) network was proposed and attempted to extract features by CNN and obtain the temporal information by LSTM layers. However, most CNN-LSTM studies use 1-D or 2-D convolutional kernels and full connected layers to process EEG data. The original EEG was transformed into 1-D or 2-D tensors, which were then fed into the LSTM layers. The above algorithms disrupt the temporal information and the transformed data does not have a real time sequence in the “time step” dimension (Xu et al., 2020). Therefore, the effect of LSTM layers is weakened because of the wrong temporal information.

### 1.2. Contribution

To fill the research gap mentioned above, in this study, a convenient and efficient end-to-end BCI framework for MWL estimation was proposed. The contributions of the article can be summarized as follows:

First, our proposed end-to-end BCI framework for workers' MWL estimation, which decodes mental workload related relevant information directly from raw EEG, is able to avoid the time consumption associated with complex feature extraction and thus meet the hardware requirements of brain-computer interface systems.

Second, this method uses a combination of filters, ASR, and ICA with ADJUST to obtain relatively pure EEG signals without manual involvement. Additionally, the MWL related neural information is decoded smoothly from the original EEG by the designed time fixed 3-D-CNN layers while the temporal dimension is unchanged. Then the following bi-LSTM layers can be used to extract temporal features.

Third, according to two types of comparison experiments and ablation studies, the estimation effectiveness of EEG-TNet can be proved.

## 2. Neural Network Preliminary

In this section, some preliminary knowledge about the neural network including convolutional layer, LSTM, and fully connected layers were introduced, which are the basis of our EEG-TNet method.

### 2.1. Convolutional Layer

In this study, convolutional layers include four normal convolutional layers, a depthwise convolutional, and a pointwise convolutional. For the normal convolutional, the input of the convolutional layer is *X*_*in*_(*C*_*in*_, *D*_*in*_, *H*_*in*_, *W*_*in*_), and the output *y*_*out*_(*C*_*out*_, *D*_*out*_, *H*_*out*_, *W*_*out*_). The formula of the convolutional layer can be described as follows:


(1) $$\begin{array}{l} y_{out}=b+\overset{C_{in}-1}{\underset{k=0}{\sum }}w⋆x_{in} \end{array}$$

Where the ⋆ is the valid 3D cross-correlation operation. The shape of *y*_*out*_(*C*_*out*_, *D*_*out*_, *H*_*out*_, *W*_*out*_) can be calculated according to the kernel size (*K*_*D*_, *K*_*H*_, *K*_*W*_) and the kernel number *C*_*out*_. Specifically, the depthwise separable convolution (Chollet, 2017) which consists of depthwise convolutional and pointwise convolutional was used in our research to extract spatial information from EEG with a lower number of convolutional parameters (Chollet, 2017).

### 2.2. LSTM Layer

By designing time-fixed 3D convolutional layers, we retain the EEG information in each time step and further analyze the temporal information using LSTM networks (Hochreiter and Schmidhuber, 1997). Recurrent neural networks (RNN) have an excellent memory capability owing to their distinctive self-connected structure, which has an absolute advantage in processing temporal data (Mikolov et al., 2010). The LSTM network is a popular expansion of RNN to address the gradient disappearance problem while RNN only processes long-term data. The LSTM introduces a gating mechanism to control the rate of accumulation of information, including adding new information and forgetting previous information by using the gates. There are three gates including input gate *i*_*t*_, forget gate *f*_*t*_, cell gate *g*_*t*_, and output gate *o*_*t*_, respectively. Specifically, the forget gate *f*_*t*_ controls the rates of previous information required to be forgotten about the internal state *c*_*t*−1_ at the last moment.


(2) $$\begin{array}{l} f_{t}=\sigma \left(W_{f}\left[h_{t-1},x_{t}\right]+b_{f}\right) \end{array}$$

The input gate *i*_*t*_ determines the rates of new information which is allowed to be added to the current *c*_*t*_. Two steps are required to achieve this. First, calculate the input gate *i*_*t*_ and cell gate *g*_*t*_ are calculated. Second, update memory cells *C*_*t*_ by combining forgetting gates *f*_*t*_ and input gates *i*_*t*_.


(3) $$\begin{array}{l} i_{t}=\sigma \left(W_{i}\left[h_{t-1},x_{t}\right]+b_{i}\right) \end{array}$$

(4) $$\begin{array}{l} g_{t}=tanh\left(W_{c}\left[h_{t-1},x_{t}\right]+b_{C}\right) \end{array}$$

(5) $$\begin{array}{l} C_{t}=f_{t}*C_{t-1}+i_{t}*g_{t} \end{array}$$

Ultimately, we need to determine the output, which is based on the state of our memory cells *C*_*t*_. First, a sigmoid layer is used to determine which parts of the memory cell state will be output. Second, the memory cell is processed through *tanh* and multiplied by *o*_*t*_.


(6) $$\begin{array}{l} o_{t}=\sigma \left(W_{o}\left[h_{t-1},x_{t}\right]+b_{o}\right) \end{array}$$

(7) $$\begin{array}{l} h_{t}=o_{t}*tanh\left(C_{t}\right) \end{array}$$

### 2.3. Fully Connected Layer

The fully connected layer serves as an “estimator” in the entire neural network structure. The procedures such as convolutional layers, pooling, LSTM, and activation function translate the original data to the hidden feature space. The fully connected layer transfers them to the sample labeling space. As Equation (8) shows, the fully connected layer multiplies the weight matrix with the input vector and then adds the bias.


(8) $$\begin{array}{l} y=xA^{T}+b \end{array}$$

where *A*^*T*^ is the learnable parameter and *b* is the bias. In addition, a softmax activation function may be used to calculate the likely distribution of the output classes. In the final FC layer, the softmax function is utilized, which is defined as follows:


(9) $$\begin{array}{l} S_{i}=\frac{e^{i}}{\sum_{j=1}^{k}e^{j}}\;for\;i=1,...k. \end{array}$$

where *i* is the input vector, the output *S*_*i*_ is between o to 1, and $\underset{i}{\sum }S_{i}=1$

## 3. Methods

**Figure 1:**
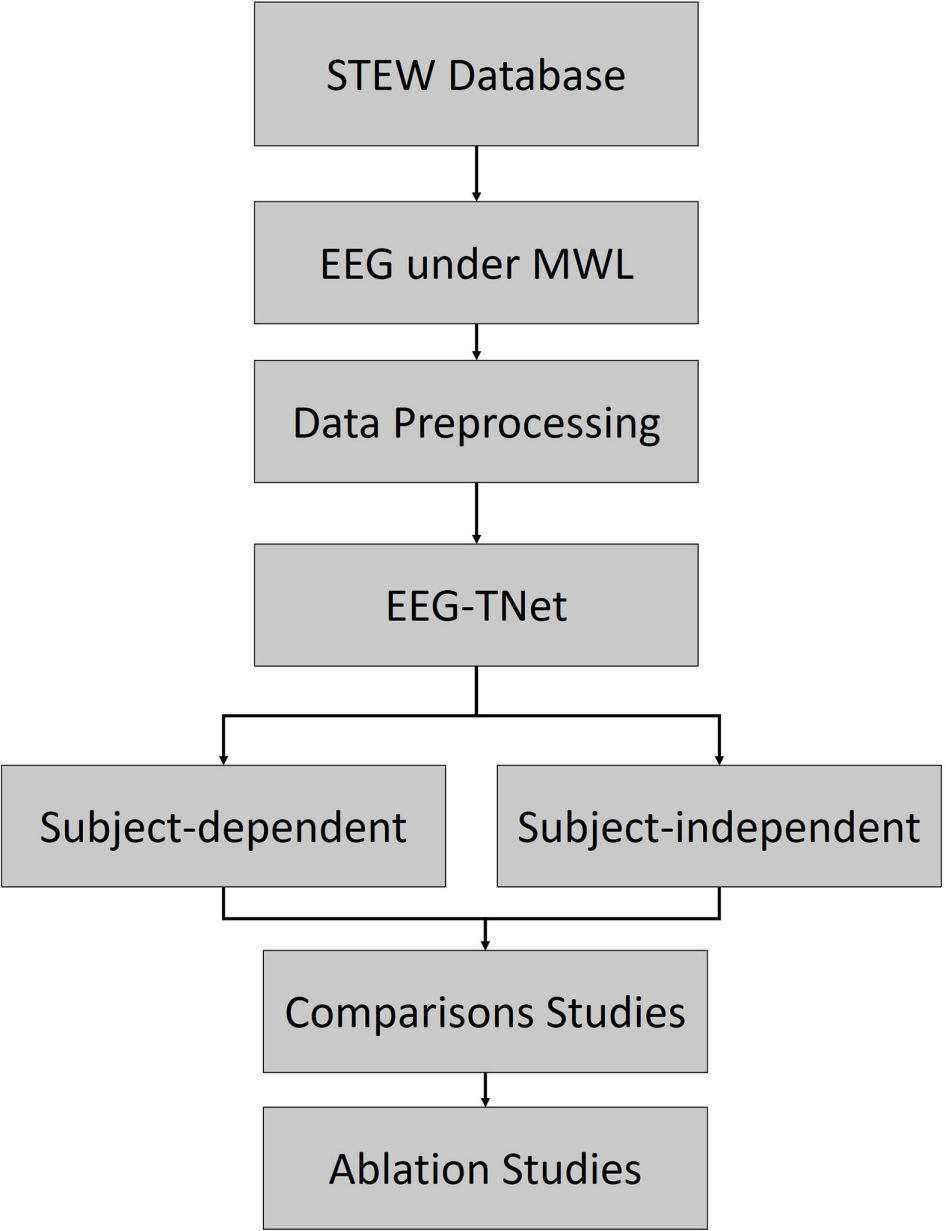
The detailed flowchart of this study.

The detailed procedure of this study can be summarized in several steps, which are described by the detailed flowchart shown in Figure 1. In this study, first, we preprocessed the data from the STEW database (Lim et al., 2018) by our designed automated methods. Then the processed EEG was directly imported to the proposed EEG-TNet to estimate the MWL. The comparison studies and ablation studies were performed to prove the effectiveness of the proposed end-to-end EEG-TNet model.

### 3.1. MWL EEG Database

The database used in this study is STEW (Lim et al., 2018), which contains EEG data of 48 subjects under different MWL levels. Specifically, the subjects performed the Simultaneous Capacity (SIMKAP) test to induce MWL. After the test, all the subjects were required to finish the subjective questionnaire to report their MWL, which is a 9-point rating scale. During the whole experiments, the EEG signals were recorded using an Emotiv EPOC EEG headset with 14 electrodes (AF3, F7, F3, FC5, T7, P7, O1, O2, P8, T8, FC6, F4, F8, AF4) and two reference channels (CMS, DRL). The sampling frequency was 128 Hz and the resolution was 16-bit A/D. In this study, the classifier was proposed to finish two tasks, the first one is classified “No Task” vs. “SIMKAP Task”, which was a binary classification. The second task was classifying Low vs. Moderate vs. High MWL, which was divided by a rating scale. A detailed definition of the label can be found in the article (Lim et al., 2018).

### 3.2. Data Preprocessing

To meet the requirements of the automation process, we eliminated parts of the preprocessing process that require manual intervention, such as manual artifact removal and manual judgment of ICA components to remove artifacts, especially eye movement artifacts (Fan et al., 2021; Peng et al., 2021). This undoubtedly reduces the quality of the data and, therefore, the accuracy of the recognition, but it makes sense for real-world applications (Rosanne et al., 2021). Table 1 shows the comparison of traditional preprocessing steps and ours. The whole preprocessing steps are

1. High-pass filter raw data at 1 Hz and low-pass filter raw data at 40 Hz.
2. Notch filter raw data at 50 Hz to avoid power line interference.
3. Perform Artifact Subspace Reconstruction (ASR) (Chang et al., 2018).
4. Perform Independent Component Analysis (ICA).
5. ADJUST (Mognon et al., 2011) was performed to automated inspect the artifact component from ICA.
6. Average re-reference the data channels.

**Table 1:** Comparison of traditional preprocessing steps and ours.

**Traditional steps**	**Human interference**	**Ours**	**Human interference**	**Goal**
Filters	NO	Filters	NO	Remove low-frequency drifts and power line interference
Manual artifacts remove	YES	ASR	NO	Remove drifts like muscle activity, sensor motion
ICA with manual judgment	YES	ICA with ADJUST	NO	Remove artifacts especially eye movement artifact

### 3.3. EEG-TNet Architecture

**Figure 2:**
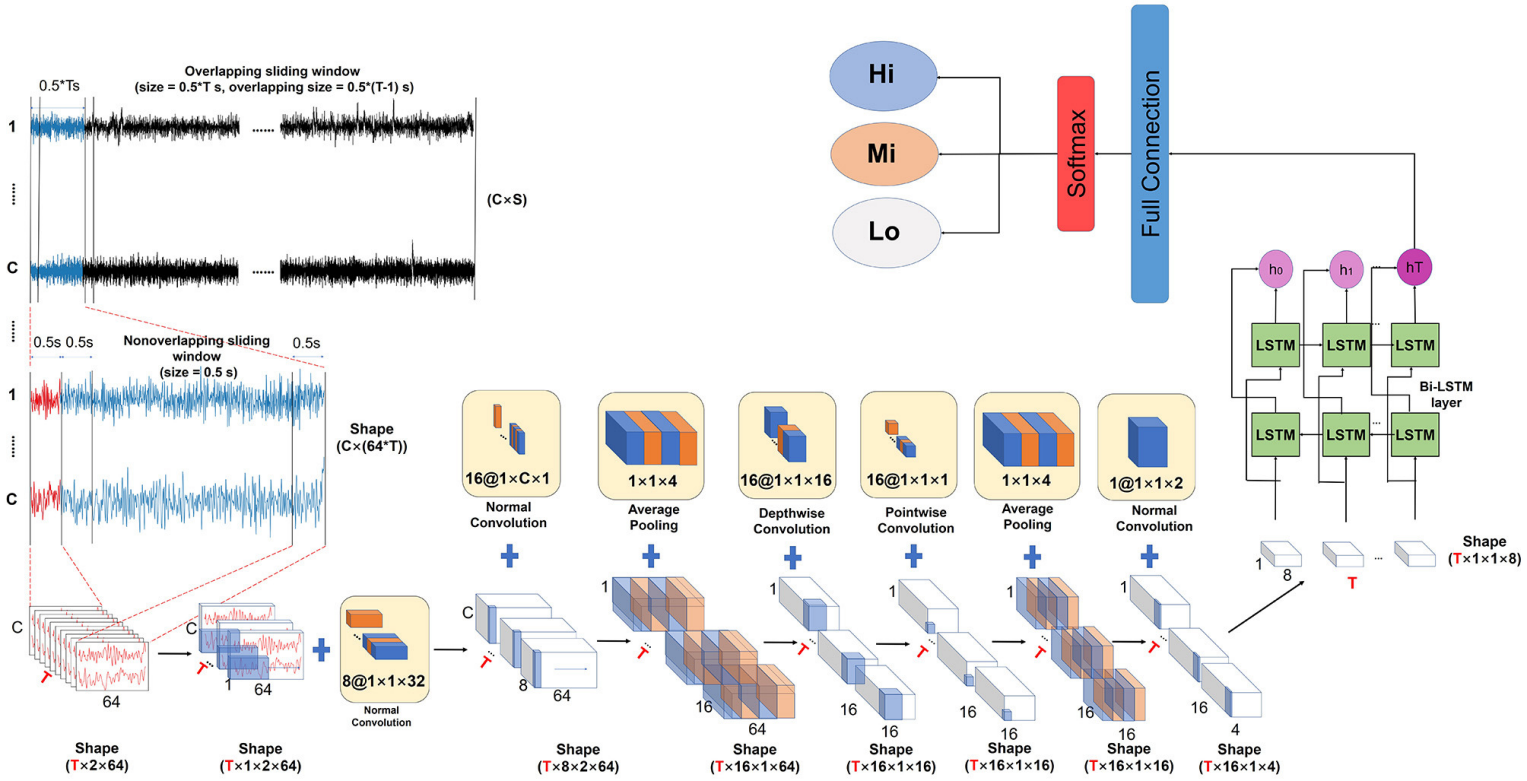
The framework of the EEG-TNet model. The EEG-TNet model consists of Data segmentation, dimension expansion, time fixed 3-D-CNN layers, Bi-LSTM layer, fully connected layers, and softmax operation.

The architecture of our EEG-TNet framework is inspired by the network architecture EEGNet of Lawhern et al. (2018), which is a widely used end-to-end EEG BCI framework. The detailed framework of our proposed EEG-TNet model can be summarized in three steps, which are shown in Figure 2. Step 1 is to segment the raw EEG to the required size and expand a new dimension for the model need, which is used to keep the temporal dimension stable. Step 2 is to extract the temporal and spatial information from each EEG fragment without between-fragments temporal information loss according to the designed temporal fixed 3-D-CNN layers. Step 3 is to extract the temporal information between each EEG fragment by using the LSTM layer. The output of the last time step in the last layer is used to compute the final status according to the fully connected layer and the softmax function.

#### 3.3.1. Data Conversion

The original EEG signals are defined as $D=\left(d_{1},d_{2},...d_{S}\right)\in {\mathbb{R}}^{S}\times C$, where *S* is the time- series length of the original EEG, and *C* denotes the channel number. Similar to the previous BCI task, the original EEG was segmented and constructed by using the overlapping sliding window and non-overlapping sliding window. The input dataset $\hat{X}\;=\;$ (${\hat{x}}_{1},{\hat{x}}_{2},...{\hat{x}}_{M}$) ∈ ℝ^*M* × *T* × *C* × *L*^, where *M* is the number of samples. The sample size of each sample ${\hat{x}}_{i}\in \hat{X}\in \left(1,2,...,M\right)$ was (*T* × *C* × *L*), the *C* × *L* is the size of per EEG fragments, which is set as 64 × 14 in this study, which means the 0.5s EEG signals of two forehead channels. In addition, the number of EEG fragments was *T*, the larger T represents the longer EEG data considered per sample. Most of the related studies analyze the input sample as a graph, where *C* × *L* is the height and width of the graph, and the dimension *T* is the channel size of EEG, such as the RGB. However, after 2-d convolutional layers, the temporal information between each fragment might be lost. In this step, we expand a new dimension whose size is 1 to meet the requirement of channel size. Furthermore, dimension *T* was considered the depth of the sample, which is stale during the whole convolutional process. Finally, the dataset $X=\left(x_{1}.x_{2}...,x_{N}\right)\in {\mathbb{R}}^{N\times T\times 1\times C\times L}$.

#### 3.3.2. Time-Fixed Convolutional Layer

Four convolutional layers were used in the EEG-TNet model. To ensure the temporal dimension is unchanged, the *K*_*D*_ of the kernel size (*K*_*D*_, *K*_*H*_, *K*_*D*_) is set as 1 during the whole convolutional processing. First, eight 3-d normal convolutional filters of size (1,1,2/L) were used to extract frequency features from the EEG signal (Lawhern et al., 2018). Then 16 convolutional filers of size (1,*C*,1) are fitted for the channel information aggregation. Subsequently, an average pooling operation (kernel size = 1 × 1 × 4) is performed to aggregate information and reduce the data dimension. Then, 16 depthwise separable convolutions are constructed, which consists of 16 depthwise convolutional filters (1 × 1 × 16) and pointwise convolutional filters (1 × 1 × 1).

#### 3.3.3. Bi-LSTM Layer

As we introduced before, the traditional LSTM layer receives the inputs solely in the forward direction through hidden states, which only retains the past information. Bidirectional LSTM (BLSTM) has been proposed to solve the problem, which has two layers named forward layer and backward layer. The forward layer is computed forward from moment 1 to moment *t*, and the output of the forward hidden layer is obtained and saved at each moment. In the backward layer, the output of the backward implicit layer is obtained and stored at each moment by computing the backward layer from moment *t* to moment 1. The final output is obtained at each moment by combining the output of the forward and backward layers at the corresponding moment. In this study, as shown in Figure 2, the output sample shape of the time-fixed convolutional layers is *T* × 1 × 1 × 8, so that both the forward layer and backward of Bi-LSTM have 8 cells to fill the data shape.

## 4. Results

In this section, two types of experiments were conducted to evaluate the MWL estimation performance using the proposed EEG-TNet BCI framework. The first type of experiment is subject-dependent while the second one is subject-independent.

### 4.1. Subject-Dependent Experiment

In the subject-dependent experiment, we adopt a similar experimental protocol as that of Chakladar et al. (2020); Kingphai and Moshfeghi (2021); Zhu et al. (2021). The five-fold cross-validation method was applied to evaluate the performance of the framework. As shown in Figure 2, specifically, the first 80% of all samples were selected as the training set and the remaining 20% of the samples were kept aside as the test dataset. Then the other 20% of the samples were selected as the second test set, the last 80% of samples were the second training set. Divide all samples like this five times and the average accuracy of the five experiments was taken as the final result. In addition, five-fold cross-validation was also applied to find optimal hyperparameters.

The proposed EEG-TNet framework was compared with the other four baseline methods for MWL estimation on the STEW dataset under the subject-dependent experiments setting, as shown in Table 2. The results showed that the proposed EEG-TNet framework achieved higher estimation accuracies than the other four methods. Although most of the recent studies use many kinds of features like frequency features [PSD (Chakladar et al., 2020; Kingphai and Moshfeghi, 2021)], non-linear features [Approximate Entropy (ApEn) (Chakladar et al., 2020; Kingphai and Moshfeghi, 2021)], linear features [autoregressive coefficient (AR) (Chakladar et al., 2020)], and the graph-based features (clustering coefficient, mean degree) (Zhu et al., 2021). However, traditional machine learning models (SVM and random forest cannot learn the full EEG information. Moreover, some combined deep neural networks (CNN-LSTM, BLSTM-LSTM) show better performance than machine learning models, it is still well below our proposed model.

**Table 2:** Comparisons of the estimation accuracy (%) of subject-dependent experiments among the various methods.

**References**	**Method**	**Features**	**No task vs. task**	**Lo vs. Mi vs. Hi**
Zhu et al. (2021)	SVM	Graph features	89.60	79.50
Chakladar et al. (2020)	Random Forest	Frequency features Linear features Non-Linear features	83.00	78.46
Chakladar et al. (2020)	CNN-LSTM	Frequency features Linear features Non-Linear features	85.21	76.76
Kingphai and Moshfeghi (2021)	BLSTM-LSTM	Frequency features Non-Linear features	91.15	89.44
	**EEG-TNet**	None	**99.82**	**99.21**

*The bold values indicate the best performance accuracies*.

### 4.2. Subject-Independent Experiment

**Figure 3:**
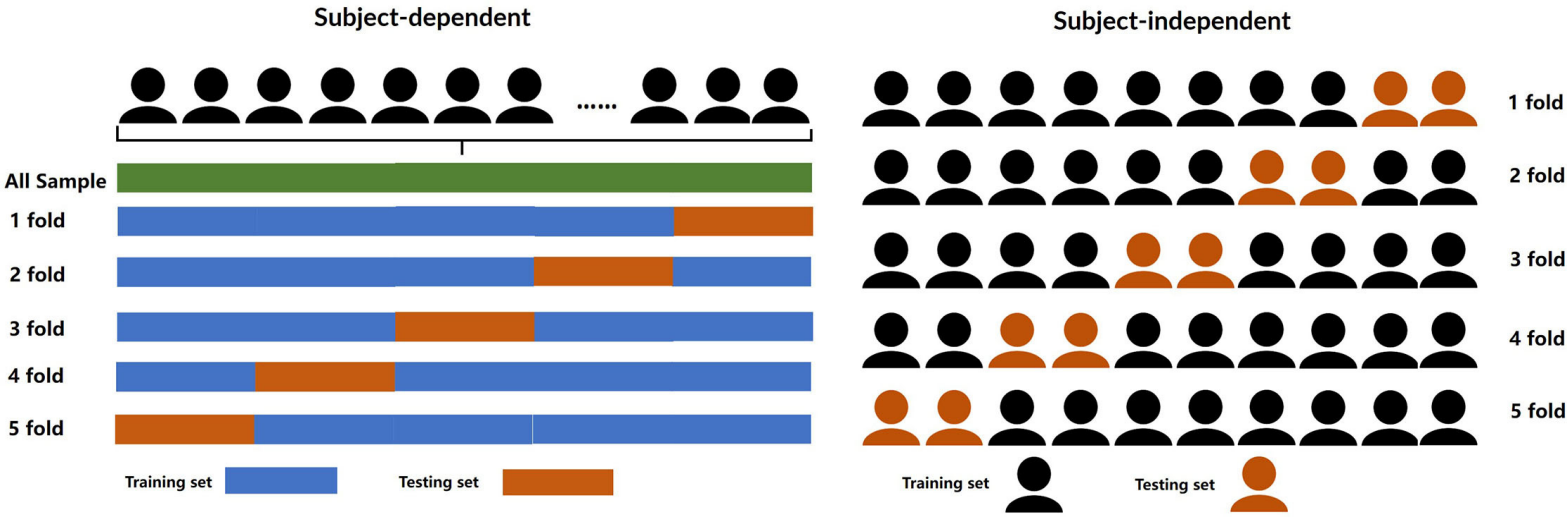
The divided methods of different experiments.

The leave-subject-out (LSO) cross-validation method was used to evaluate the performance of the proposed framework in the subject-independent experiments. As shown in Figure 3, in the LSO cross-validation experimental protocol, the EEG samples of 36 subjects (80% of a total of 45 subjects) were selected for training the model and the last EEG samples of 8 subjects (20% of a total of 45 subjects) were used to test the model performance. The whole process was repeated five times so that all the subjects' samples were taken as the test set. The average accuracy of the five experiments was taken as the final result. Similarly, the hyperparameters were found according to the five-fold cross-validation in the training set.

Compared with the subject-dependent experiments, there are fewer studies that perform the subject-independent experiments because of their difficulty. The proposed EEG-TNet framework was compared with the other three baseline methods under the subject-independent experiments setting, as shown in Table 3. It is worth noting that no studies were conducted with either dual-task estimation (No Task vs. Task) or triple task estimation (Lo vs. Mi vs. Hi) subject-independent experiments simultaneously until now (Lim et al., 2018; Pandey et al., 2020).

**Table 3:** Comparisons of the estimation accuracy (%) of subject-independent experiments among the various methods.

**References**	**Method**	**Features**	**No task vs. task**	**Lo vs. Mi vs. Hi**
Pandey et al. (2020)	KNN	None	61.08	\
Pandey et al. (2020)	MLP	None	58.68	\
Pandey et al. (2020)	LSTM	None	57.30	\
Lim et al. (2018)	SVM	Frequency features	\	**69.00**
	**EEG-TNet**	None	**82.78**	66.83

*The bold values indicate the best performance accuracies*.

Table 3 summarized the comparative results regarding the average estimation accuracy under the subject-independent experiments setting. Pandey et al. (2020) realized that handcrafted features would slow down the speed of computing and significantly increased the evaluation time, making it challenging to apply them in practical scenarios. Therefore, he used an end-to-end structure similar to ours to finish the dual-task estimation(No Task vs. Task). However, the results were unsatisfactory, with his best assessment only reaching 61.08%, which is only a tiny improvement over the random classification (50%).

**Figure 4:**
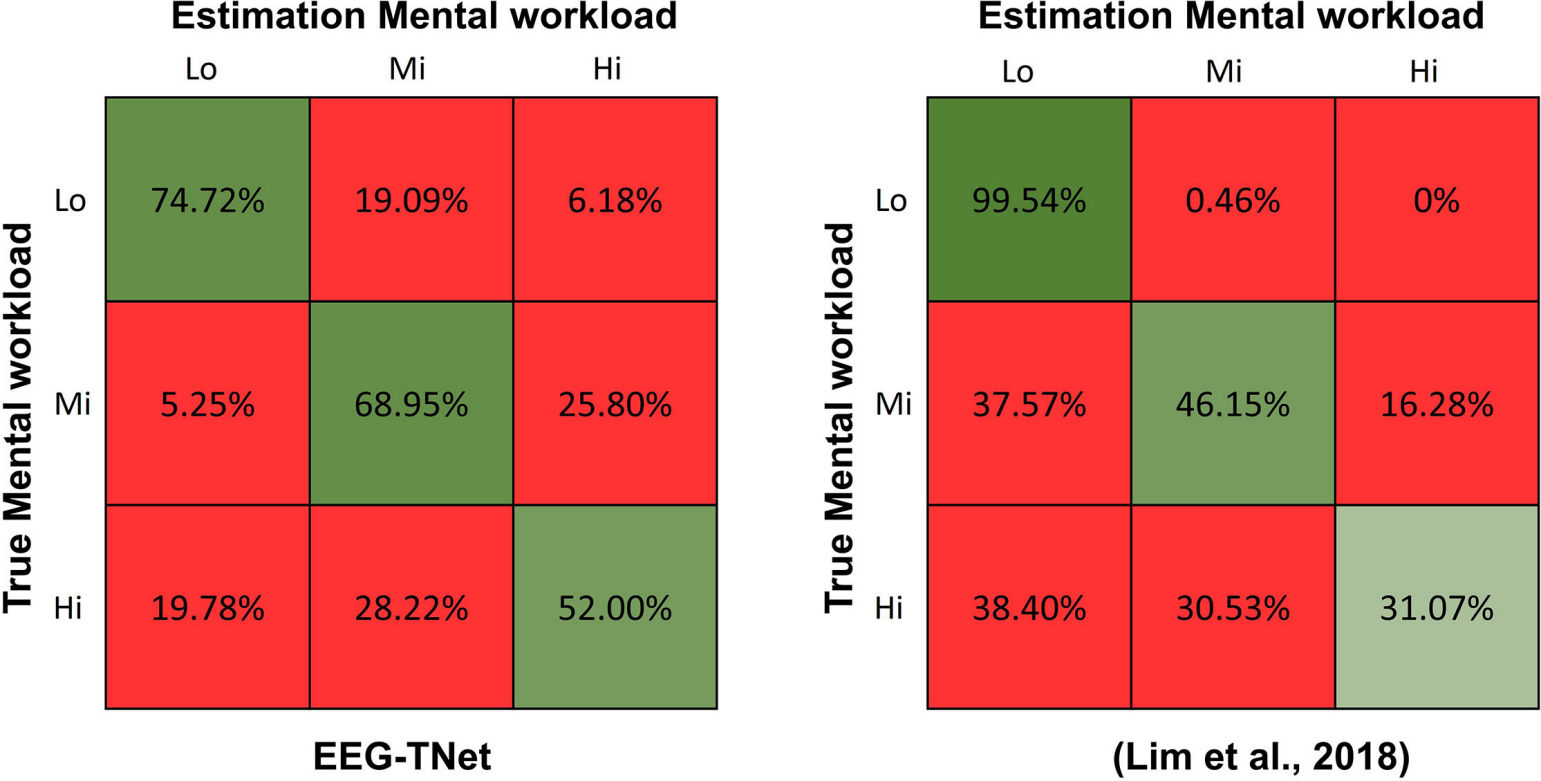
Confusion matrix of EEG-TNet and Lim's work.

For practical reasons, we need to focus more on triple-task estimation (Lo vs. Mi vs. Hi) than on the dual-task estimation (No Task vs. Task). Lim et al. (2018) contributed the STEW dataset, where he extracted the PSD of different bans as features and used SVM as a classifier. Although their recognition results were slightly higher than our results, comparing the confusion matrices shows that our estimation results are more balanced and valid. As Figure 4 shows, the accuracy of their results was 99.54% for low MWL, down to 46.15% for medium workloads and only 31.07% for high MWL, which was even lower than the random results (33.33%). In contrast, our estimation accuracies range from 52.00 to 74.72%. On the most difficult estimation task with high MWL, our results were nearly twice as good as theirs.

## 5. Discussion

### 5.1. Practicability

The main objective of this study is to propose a practical and effective MWL estimation method for workers of special occupational groups, which can be used to ensure safety during the course of their work. EEG signals are regarded as the gold standard and BCI systems based on EEG signals have natural advantages. However, most BCI systems cannot meet the requirements of online evaluation due to the high manual involvement in signal noise reduction, complex and time-consuming feature extraction, and other disadvantages. In order to solve the disadvantage of manual involvement in signal noise reduction, this system uses a combination of filters, ASR, and ICA with ADJUST to obtain relatively pure EEG signals without manual involvement.

The classification model is at the heart of the BCI system, because of the system's computing time, deployment method, and evaluation accuracy depends on it. In most related BCI systems, the handcrafted features were used to estimate the MWL, which may be computationally demanding and not suitable for a real-time system. This study exploited deep neural networks' powerful feature extraction and classification capabilities to design the EEG-TNet network as the computational core of an end-to-end BCI framework. This study improves the traditional neural network model named EEG-Net and extracts features through the processes of data segmentation, dimension expansion, time fixed 3-D-CNN layers, Bi-LSTM layer, fully connected layer, and softmax operation. The time-fixed method was designed to ensure the temporal segment order, and a Bi-LSTM layer was added at the end for temporal information analysis. Moreover, the total time cost of this model is only 386.74 ms in our machine [System: Ubuntu 20.04, CPU: Intel(R) Core(TM) i7-8700K CPU @ 3.70GHz, Memory: 32 GB, GPU: GeForce RTX 2080Ti]. The low time cost proves that the proposed EEG-TNet can meet the requirements of real-time application.

### 5.2. Estimation Performance

The most important metric for evaluating a model is estimation accuracy. Unlike other research areas, both subject-dependent experiments and subject-independent experiments need to be considered in the field of human factors engineering. In most cases, subject-dependent experiments are more accurate because such experimental methods allow the model to obtain EEG data for each individual during the training phase. Information on individual differences can be extracted. As shown in Table 2, most studies have achieved more than 80% or even more than 90% recognition accuracy, while our recognition accuracy is close to 100%.

However, the subject-dependent experimental approach is often not applicable to practical scenarios. Training a unique classification model for each worker would be time-consuming and costly, so subject-independent experiments use unseen subjects' data as the test set, satisfying the need for “Plug-and-Play”. However, head shape, scalp impedance, and psychological state can all affect the EEG data, resulting in large variations in EEG data among subjects. The accuracy of the method is poor. Therefore, as shown in Table 3, almost all of the previous methods do not apply to estimating the MWL of workers. In the dual-task estimation, the method proposed by Pandey et al. (2020) was only marginally more accurate than random. In contrast, our method was able to achieve 82.78%, which is sufficient for use in realistic scenarios.

The triple task estimation(Lo vs. Mi vs. Hi) is the most urgent from the point of view of ensuring safety in different fields like transportation and construction. Accurate assessment of the high or moderate MWL of workers helps managers to allocate tasks rationally and to avoid overloading workers with work that could lead to human factor accidents. Although the estimation accuracy of the method proposed by Lim et al. (2018) appears to be slightly higher than our proposed EEG-TNet method. By analyzing and comparing the confusion matrices of the two methods in Figure 4, the method of Lim et al. (2018) may not apply to worker workload estimation. According to their confusion matrix, we can find that the low MWL was assessed at 99.54%, which means that almost all low load situations were successfully identified. However, the estimation accuracy for moderate MWL dropped to 46.15%, and only 31.07% of the samples with high MWL were correctly estimated, with an accuracy rate even lower than the random results (33.33%). More notably, 37.57% of the medium workload samples and 38.40% of the high MWL samples were misclassified as low MWL. In many occupational fields, underestimating workers' workload by managers can lead to their scheduling of excessive workloads, leading to workers being overloaded, making the chances of unsafe behavior much higher. In our assessment results, just 5.25% of the moderately loaded and 19.78% of the high loaded sample were incorrectly underestimated as low workloads. Compared to Lim et al. (2018), the likelihood of underestimation was 7 times and 2 times lower, respectively.

**Figure 5:**
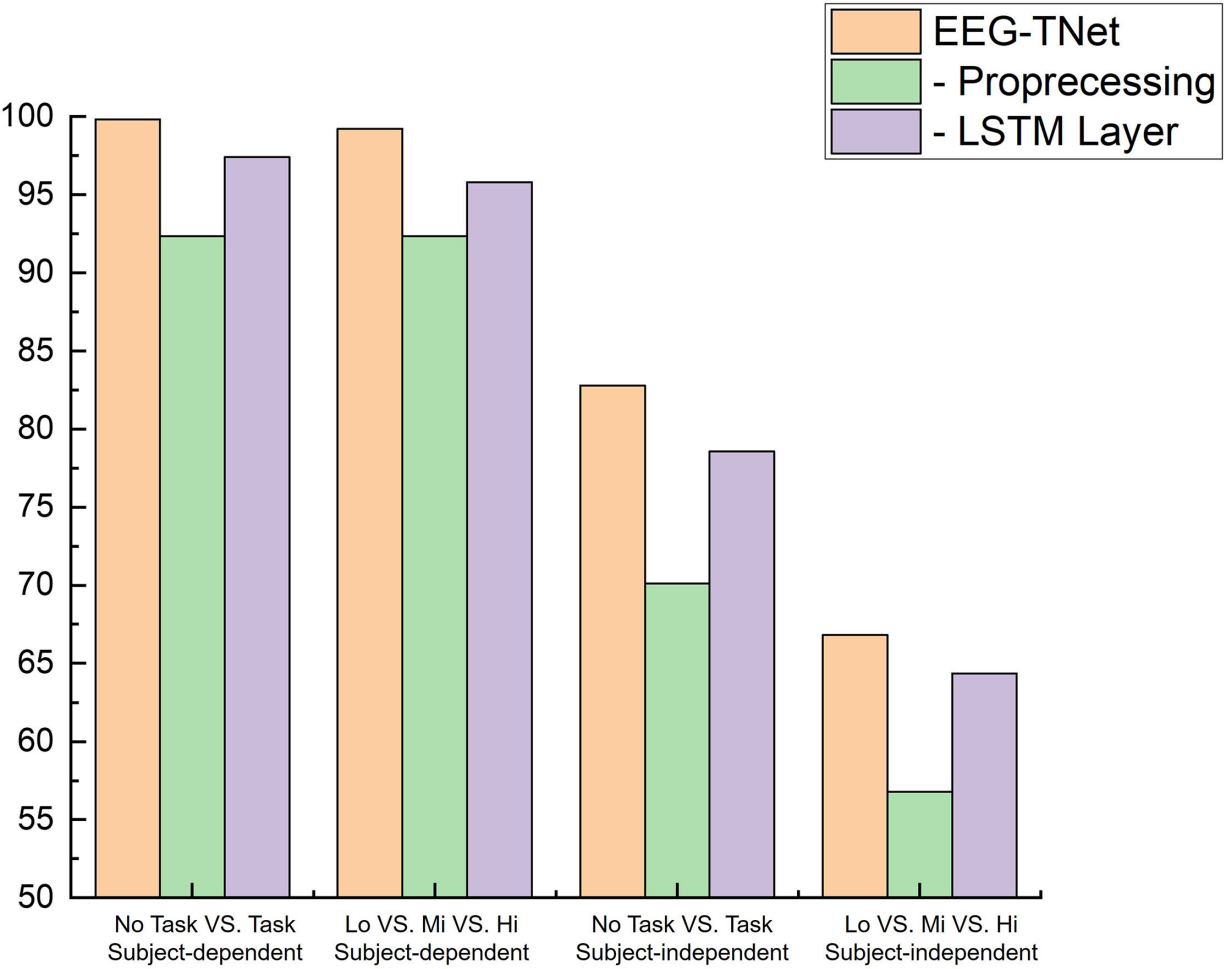
Ablation studies.

To verify the effectiveness of our model, ablation experiments are performed on the STEW database. There are two kinds of ablation experiments: (1) Ablation experiments on the effectiveness of designed automated preprocessing method (2) Ablation experiments on the effectiveness of Bi-LSTM. As Figure 5 shows, estimation accuracy significantly decreased when the data pre-processing process or LSTM layer was removed, not only in subject-dependent experiments but also in subject-independent experiments.

However, there were some limitations to this study. First, this study used the multi-channel EEG to build the EEG-TNet model. However, collecting multi-channel EEG needs gel-based EEG caps or clumsy dry electrode EEG caps, which is too troublesome to fill the practical usage. Additionally, the increase in the number of channels also brings a significant increase in computational complexity. Furthermore, although the estimation accuracy for high MWL is much larger than the previous study, it is still not enough for application scenarios. Finally, the STEW database only contains 48 students' EEG data under different levels of MWL, which were not selected to be representative. It is important to analyze the EEG signals of different occupational groups, ages, and work experiences.

## 6. Conclusion

This study proposed an end-to-end BCI framework named EEG-TNet for the estimation of worker MWL using EEG signals and conducted different types of experiments to assess the effectiveness of the EEG-TNet framework. In the subject-dependent experiments, the estimation accuracy of dual-task estimation (No task vs. TASK) and that of triple-task estimation (Lo vs. Mi vs. Hi) reach 99.82 and 99.21% respectively. Compared with the state-of-the-art methods proposed in previous studies, the accuracy is improved by 8.67 and 9.77%, respectively. Although there is a substantial decrease in estimation accuracy in subject-independent experiments, the accuracy of different tasks still reaches 82.78 and 66.83% respectively. Especially, in the subject-independent experiments, compared to previous study, the likelihood of underestimation was 7 times and 2 times lower respectively, which means that our proposed EEG-TNet model can fill the requirement of real-time application. In the future, we will extend the research by designing new network structures such as graph neural networks (GNN) to improve the estimation accuracy of high MWL and designing a closed-loop system that includes real-time estimation and feedback systems. Additionally, building a new database that includes more occupational groups will also be our future direction.

## Data Availability Statement

The datasets presented in this study can be found in online repositories. The names of the repository/repositories and accession number(s) can be found below: https://ieee-dataport.org/open-access/stew-simultaneous-task-eeg-workload-dataset.

## Author Contributions

CF responsible for the conceptualization, performed the majority of the experiments and analyzes, made the figures, and wrote the first draft of the manuscript. JH and SH performed some experiments, updated the figures, performed the statistics, and edited the manuscript. YP responsible for the methodology, project administration, resources, funding acquisition, and validation. SK responsible for the conceptualization and supervision. All authors contributed to the article and approved the submitted version.

## Funding

This study was supported by the National Natural Science Foundation of China [Grant Numbers: 52075553]; the Natural Science Foundation of Hunan [Grant Numbers: 2020JJ7030]; the Hunan Science Foundation for Distinguished Young Scholars of China [Grant Numbers: 2021JJ10059]; the Postgraduate Scientific Research Innovation Project of Hunan Province [Grant Numbers: CX20210099].

## Conflict of Interest

JH was employed by the Hunan Communications Research Institute Co., Ltd., Hunan Communication & Water Conservancy Group Ltd. The remaining authors declare that the research was conducted in the absence of any commercial or financial relationships that could be construed as a potential conflict of interest.

## Publisher's Note

All claims expressed in this article are solely those of the authors and do not necessarily represent those of their affiliated organizations, or those of the publisher, the editors and the reviewers. Any product that may be evaluated in this article, or claim that may be made by its manufacturer, is not guaranteed or endorsed by the publisher.
